# Comparison of Diversion Strategies for Management of Acute Complicated Diverticulitis in a US Nationwide Cohort

**DOI:** 10.1001/jamanetworkopen.2021.30674

**Published:** 2021-11-05

**Authors:** Yas Sanaiha, Joseph Hadaya, Esteban Aguayo, Formosa Chen, Peyman Benharash

**Affiliations:** 1 Cardiac Outcomes Research Laboratory, University of California, Los Angeles; 2 Department of Surgery, David Geffen School of Medicine at the University of California, Los Angeles, Sylmara

## Abstract

**Importance:**

Diverticulitis of the colon is an increasingly prevalent disease with significant implications for patient quality of life and health system resource expenditure. Although several randomized clinical trials and meta-analyses of Hartman procedure (HP) and primary anastomosis and proximal diversion (PAPD) have found surgical equipoise, questions regarding the relative performance of these treatments when applied broadly remain.

**Objective:**

To examine use of and outcomes after urgent sigmoid colectomy with end colostomy (ie, HP) vs PAPD in management of complicated diverticulitis.

**Design, Setting, and Participants:**

This retrospective cross-sectional study was a multicenter, population-based examination of inpatient hospitalizations, not including long-term rehabilitation facilities, using data from the 2014 to 2017 Nationwide Readmissions Database. It was performed from November 2020 to January 2021. Included patients were adults admitted with acute diverticulitis requiring HP or PAPD within 48 hours of admission.

**Exposures:**

Undergoing HP vs PAPD.

**Main Outcomes and Measures:**

Inverse probability treatment analysis was used to compare outcomes, including index mortality, composite complications (ie, neurologic, infectious, and cardiovascular complications), length of stay, and readmissions within 90 days.

**Results:**

During the study period, an estimated 1 072 395 adults (615 954 [57.4%] women; median [IQR] age, 64 [52-76] years) required nonelective hospitalization for acute colonic diverticulutus. A total of 34 126 patients required diversion, with 32 326 patients (94.7%) undergoing HP and 1800 patients (5.3%) undergoing PAPD within 48 hours of admission. Patients undergoing PAPD had a decreased median (IQR) age (60 [51-70] years vs 65 [54-74] years; *P* < .001) and decreased rates of end organ dysfunction (520 patients [28.9%] vs 11 514 patients [35.6%]; *P* < .001). In inverse probability treatment weight analysis, the odds of mortality (adjusted odds ratio [aOR], 0.63; 95% CI, 0.32-1.40), complications (aOR, 0.86; 95% CI, 0.66-1.13), and nonhome discharge (aOR 1.15; 95% CI, 0.83-1.60) were similar for PAPD compared with HP. Among 1772 patients who underwent PAPD and survived index hospitalization, there was an increased incidence of 90-day readmission compared with 30 851 patients who underwent HP and survived index hospitalization (393 patients [22.2%] vs 4384 patients [14.2%]; *P* < .001) with increased hazard of ostomy reversal (hazard ratio, 1.46; 95% CI, 1.08-1.99).

**Conclusions and Relevance:**

This study found that the use of PAPD was associated with comparable index hospitalization outcomes vs use of HP, while readmission rate and ostomy risk were statistically significantly increased among patients who underwent PAPD compared with patients who underwent HP. These findings suggest that further delineation of criteria for appropriate application of PAPD in the urgent setting are warranted.

## Key Points

**Question:**

Among patients with acute diverticulitis treated with an urgent surgical procedure (ie, Hartmann procedure [HP] or primary anastomosis with proximal diversion [PAPD]), is the procedure used associated with index readmission outcomes and resource use?

**Findings:**

In this cross-sectional study of an estimated 1 072 395 hospitalized patients with acute diverticulitis, 34 126 patients required diversion within 48 hours of admission, and PAPD was associated with comparable index outcomes vs HP but an increased rate of readmission and risk of ostomy reversal. Total index and readmission hospitalization costs were similar between procedures.

**Meaning:**

These findings suggest that PAPD is associated with increased risk of ostomy reversal compared with HP and that patient characteristics that bias operative strategy selection are the primary factors associated with index patient outcomes.

## Teaser

This cross-sectional study compares adverse outcomes and health care use associated with primary anastomosis with proximal diversion vs Hartmann procedure for patients with acute diverticulitis.

## Introduction

With an increasing burden in industrialized nations, diverticulitis has been found to be associated with 1.5 million days of inpatient care and more than 2 billion dollars in annual hospitalization costs across the US.^1^ The urgent surgical management of acute perforated diverticulitis has significantly evolved, and the 2-stage operation known as Hartmann procedure (HP) is currently the most common approach.^2,3,4^ More recently, use of primary anastomosis with or without diverting loop ileostomy (PAPD) has been suggested to be safe in the treatment of patients with gross peritoneal contamination.^5,6^

To date, several randomized clinical trials and meta-analyses have found comparable mortality and complication rates between PAPD and HP for Hinchey stage III to IV perforated diverticulitis, with reports of increased rates of stoma reversal in the PAPD group.^7,8,9,10,11,12,13^ In an attempt to evaluate the relative merits of PAPD at the national level, Gawlick et al^6^ used National Surgical Quality Improvement Program (NSQIP) data and reported similar outcomes for the 2 diversion strategies. However, significant constraints, including low NSQIP participation among hospitals, limit the extrapolation of their findings. Goldstone et al^14^ examined statewide data and found increased mortality among patients undergoing PAPD, concluding that recommendations regarding primary anastomosis in the urgent setting may need reevaluation.

In this population-based study of US hospitalizations for acute diverticulitis, we examined the association of urgent HP or PAPD with index mortality, complications, hospitalization costs, and unplanned readmission rates within 90 days of discharge. We hypothesized that the approaches would be associated with comparable short-term outcomes after risk adjustment. We further hypothesized that PAPD would be associated with increased rates of readmission, inpatient hospitalization costs, and ostomy reversal.

## Methods

The institutional review board at the University of California, Los Angeles, deemed this cross-sectional study exempt from full review and informed consent because the Nationwide Readmissions Database (NRD) contains publicly available deidentified patient data. The Strengthening the Reporting of Observational Studies in Epidemiology (STROBE) reporting guideline was followed for this study.

Nationally weighted data for all adult patients (ages ≥18 years) with nonelective hospitalization for acute colonic diverticulitis were abstracted from the 2014 to 2017 NRD. Maintained by the Agency for Healthcare Research and Quality,^15^ the NRD is the largest publicly available, all-payer database and accrues individual state inpatient data. Using a unique patient identifier, index and readmission hospitalizations are linked for the entirety of each calendar year at NRD participating institutions. *International Classification of Diseases, Ninth Revision* (*ICD-9*) and *International Statistical Classification of Diseases and Related Health Problems, Tenth Revision* (*ICD-10*) coding and unique patient linkage identifiers allow for evaluation of multiple hospitalizations in each calendar year. The NRD reports patient demographics; hospital characteristics, such as number of beds and teaching status; and resource use, including length of stay and charges.

All clinical variables were defined according to Healthcare Cost Utilization Project standards.^16^ Facilities in the highest tertile of underinsured patients across all diverticulitis admissions each year were considered safety net hospitals.^17^ The presence of multiorgan dysfunction was characterized using a combination of diagnosis codes, including hypotension and acute kidney failure, which has been previously described for assessment of patient acuity in administrative databases (eTable 1 in the Supplement).^18,19^ Hospitals in the top tertile were classified as high volume for diverticulitis admissions or diverticulitis-associated sigmoidectomy. The operative cohort was further stratified into HP and PAPD for comparative analyses using appropriate *ICD-9* and *ICD-10* procedural codes (eTable 2 in the Supplement). Patients with operative intervention later than 48 hours after admission, the diagnosis of small bowel diverticulitis, or missing data on mortality, hospitalization duration, costs, or operative procedure day were excluded (eFigure in the Supplement).

The primary outcome was index mortality after sigmoidectomy. Diversion strategy after sigmoidectomy was the applied exposure variable. A composite complication variable was defined as the presence of at least 1 of these conditions: stroke, cardiac arrest, pneumonia, pulmonary edema, acute respiratory failure, mechanical ventilation, deep vein thrombosis, pulmonary embolism, wound infection, or sepsis or septicemia (eTable 1 in the Supplement). Hospitalization costs were derived by applying hospital-specific cost-to-charge ratio files to overall charges and were inflation adjusted to the 2017 Personal Health Care Index.^20^ Several secondary outcomes, including duration of index hospitalization, ostomy reversal during the remaining months of each calendar year, and readmission within 30 days and 31 to 90 days of index discharge, were considered.

### Statistical Analysis

All statistical analyses were performed using Stata statistical software version 16 (StataCorp). Data were analyzed from November 2020 through January 2021. Statistical significance was designated at α < .05 or when 95% CIs did not cross a reference of 1. We used χ^2^ analysis of survey-weighted data and adjusted Wald 2-tailed *t* tests to compare patient and hospital characteristics. Significance of temporal trends was assessed using Royston parametric test (*P* for trend).^21^ To mitigate selection bias, we used inverse probability of treatment weights (IPTW), which were based on a propensity score accounting for patient age, sex distribution, baseline comorbidities, insurance payer, income quartile, presence of multiorgan dysfunction, presence of peritonitis, annual hospital volume of diverticulitis sigmoidectomy, and safety net status. The IPTW was used to modify the NRD-provided discharge-level weight (mean treatment association) and generate a new weight for survey-weighted analyses.^22^ Appropriate cohort balance was confirmed with univariate comparisons of baseline characteristics using IPTW discharge weight for survey estimates (eTable 3 in the Supplement). Similarly, IPTW discharge weight was used for multivariable logistic regression examining primary outcomes. Annual institutional emergency general surgical (EGS) volume was calculated, and hospitals were stratified by observed to expected ratio of EGS mortality. Hospitals outperforming their peers with observed to expected ratio greater than 2 were categorized as high performing based on the inflection point of the observed to expected ratio curve. Royston and Parmar flexible parametric models were used to assess for time-varying hazard of readmission and ostomy closure over the duration of surveillance within the constraints of the NRD.^23^ Counterfactual modeling of inpatient costs was performed to estimate the associated costs if all patients were to undergo PAPD by applying the median PAPD hospitalization costs to all patients who underwent HP.

## Results

Over the study period, an estimated 1 072 395 adults (615 954 [57.4%] women; median [IQR] age, 64 [52-76] years) required nonelective hospitalization for acute colonic diverticulitis, including 252 975 patients in 2014 and 272 216 patients in 2017, for a decrease in incidence of 1856 patients to 1635 patients per 100 000 adult hospitalizations from 2014 to 2017 (*P* for trend <.001). While the proportion of patients requiring same-admission sigmoidectomy increased from 20 490 patients (8.1%) in 2014 to 27 222 patients (10.0%) in 2017, use of diversion among these patients (ie, HP or PAPD) decreased from 13 339 patients (65.1%) in 2014 to 17 558 patients (64.5%) in 2017 (*P* for trend < .001). Among 34 126 patients in the study requiring diversion, 32 326 patients (94.7%) underwent HP and 1800 patients (5.3%) underwent PAPD, with 29 246 patients (85.7%) requiring an operation within the first 24 hours after admission. Overall, the rate of PAPD among all patients who required diversion increased from 185 of 7203 patients (2.6%) to 756 of 10 712 patients (7.1%) over the study period (*P* for trend < .001).

Compared with patients undergoing HP, patients undergoing PAPD were younger (median [IQR] age, 60 [51-70] years vs 65 [54-74] years; *P* < .001) and had decreased rates of comorbidities, including heart failure, coronary artery disease, and chronic pulmonary obstructive disease, demonstrating an overall lower risk profile. Furthermore, patients undergoing PAPD had lower rates of multiorgan dysfunction (520 patients [28.9%] vs 11 514 patients [35.6%]; *P* < .001), coagulopathy, and peritonitis (Table 1). The prevalence of diabetes and chronic liver disease was similar across the 2 operative strategies. Although there was no statistically significant difference in income distribution between groups, individuals in the PAPD group were more commonly privately insured (Table 1).

**Table 1.** Patient and Hospital Characteristics

Characteristic	Patients, No. (%)		*P* value
	Undergoing HP (n = 32 326)	Undergoing PAPD (n = 1800)	
**Patient characteristic**			
Age, median (IQR), y	65 (54-74)	60 (51-70)	<.001
Sex			
Women	16 889 (52.2)	897 (49.8)	.21
Men	15 437 (47.8)	903 (50.2)	
Congestive heart failure	3077 (9.5)	99 (5.5)	.001
Coronary artery disease	3826 (11.8)	152 (8.4)	.001
Chronic pulmonary parenchymal disease	5925 (18.3)	232 (13.0)	<.001
Pulmonary hypertension	1876 (5.8)	132 (7.3)	.11
Peripheral vascular disease	1380 (4.3)	83 (4.6)	.61
Hypertension	16 394 (50.5)	874 (49.1)	.45
Diabetes	4305 (13.3)	244 (13.6)	.86
Chronic kidney dysfunction	2724 (8.4)	89 (4.9)	<.001
Chronic liver disease	1173 (3.6)	68.8 (3.8)	.78
Coagulopathy	1816 (5.6)	51 (2.8)	<.001
Multiorgan dysfunction	11 514 (35.6)	520 (28.9)	<.001
Obesity	5073 (15.7)	299 (16.6)	.52
Weight loss	4475 (13.8)	236 (13.1)	.59
Electrolyte abnormalities	14 370 (44.5)	698 (38.8)	.004
Peritonitis	9.292 (28.7)	242 (13.4)	<.001
Ascites	1243 (3.8)	74 (4.1)	.71
Chronic steroid use	847 (2.6)	26 (1.4)	.02
Percutaneous drain	1350 (4.2)	83 (4.6)	.54
Laparoscopy	748 (2.3)	37 (2.1)	.64
Laparoscopic converted to open approach	851 (2.6)	90 (5.0)	.001
Income quartile			
0-25th	7859 (24.7)	394 (22.2)	.19
25th-50th	8891 (27.9)	491 (27.7)	
50th-75th	8311 (26.1)	445 (25.2)	
75th-100th	6831 (21.4)	441 (24.9)	
Private insurer	11 040 (34.2)	795 (44.2)	<.001
**Hospital characteristic**			
Teaching status			.36
Metropolitan, nonteaching	10 449 (32.3)	548 (30.4)	.16
Metropolitan, teaching	18 243 (56.4)	1073 (59.6)	
Rural	3634 (11.2)	179 (9.9)	
Safety net hospital status	8464 (26.2)	486 (27.0)	.68
Interhospital transfer	480 (1.5)	23 (1.3)	.65
Annual sigmoidectomy volume, median (IQR), No. operations performed	26 (14-44)	34 (18-57)	<.001
Annual diverticulitis volume, median (IQR), No. hospital admissions	150 (89-232)	153 (95-241)	.25

Abbreviations: HP, Hartmann procedure; PAPD, primary anastomosis and diverting loop ileostomy.

Use of PAPD was not associated with hospital bed capacity, teaching status, or safety net status (Table 1). Among patients receiving PAPD, 1544 patients (85.8%) received care at hospitals at the highest tertile of sigmoidectomy volume, compared with 25 731 patients receiving HP (79.6%) (*P* = .001). Annual institutional volume of diverticulitis admissions was similar across HP and PAPD groups (Table 1). Furthermore, patients undergoing PAPD less commonly received care at institutions considered high performers for mortality after emergency general surgical treatment (54 patients [3.0%] vs 1584 patients [4.9%]; *P* < .001). This association persisted in risk-adjusted models, with decreased odds of PAPD at high-performing hospitals (adjusted odds ratio [aOR], 0.52; 95% CI, 0.45-0.61).

In unadjusted comparison of outcomes between groups, there were decreased rates of index mortality (28 patients [1.5%] vs 1475 patients [4.6%]; *P* < .001), composite complications (634 patients [35.2%] vs 14 348 patients [44.4%]; *P* < .001), and nonhome discharge (332 of 1772 patients who survived to discharge [18.8%] vs 8394 of 30 851 patients who survived to discharge [27.3%]; *P* < .001) with PAPD (Table 2). Patients who received the operation within 24 hours of admission were less likely to undergo PAPD (aOR, 0.59; 95% CI, 0.49-0.71). Among 1772 patients who underwent PAPD and survived index hospitalization, there was an increased incidence of 90-day readmission compared with 30 851 patients who underwent HP and survived index hospitalization (393 patients [22.2%] vs 4384 patients [14.2%]; *P* < .001). In multivariable analysis after application of IPTW, there were no statistically significant differences in odds of mortality (aOR 0.63, 95% CI 0.32-1.40), complications (aOR 0.86, 95% CI 0.66-1.13), or nonhome discharge (aOR 1.15, 95% CI 0.83-1.60) among patients who underwent PAPD compared with those who underwent HP. Compared with patients who underwent HP, index hospitalization costs for patients who underwent PAPD were increased by $5183 (95% CI, $1270-$9097). In a sensitivity analysis of patients receiving care at hospitals with a minimum of 50 emergency general surgical operations, controlling for observed to expected mortality per institution, the odds of mortality (aOR, 0.40; 95% CI, 0.20-0.80) and complications (aOR, 0.73; 95% CI, 0.57-0.93) were decreased with PAPD.

**Table 2.** Univariate Index and Readmission Outcomes

Outcome	Patients, No. (%)^a^		*P* value
	Undergoing HP	Undergoing PAPD	
Final study cohort, No.	32 326	1800	NA
Mortality	1475 (4.6)	28 (1.5)	<.001
Composite complication	14 348 (44.4)	634 (35.2)	<.001
Stroke	82 (0.3)	NA	NA
Myocardial infarction	249 (0.8)	NA	NA
Cardiac arrest	249 (0.8)	NA	NA
Pneumonia	623 (1.9)	24 (1.4)	.18
Pulmonary embolism	319 (1.0)	15(0.9)	.67
Acute respiratory failure	4555(14.1)	160 (9.0)	<.001
Pulmonary edema	146 (0.5)	NA	NA
Deep vein thrombosis	2013 (0.6)	NA	NA
Prolonged ventilation^b^	1667 (5.2)	55 (3.0)	.005
Urinary tract infection	2101 (6.5)	108 (6.0)	.61
Surgical site infection	1125 (3.5)	78 (4.3)	.24
Sepsis	10 222 (31.6)	444 (24.7)	.0001
Septicemia	9784(30.3)	396 (22.0)	<.001
Hospitalization cost, median (IQR), $	24 139 (17 302-36 396)	25 657 (18 082-36 362)	.01
Hospitalization duration, median (IQR), d	9 (7-12)	8 (6-12)	.07
Patients surviving index hospitalization, No.	30 851	1772	NA
Nonhome discharge	8394 (27.3)	332 (18.8)	<.001
At 30 d			
Readmission	3416 (11.1)	336 (19.0)	<.001
Readmission mortality	159 (4.6)	NA	NA
Cost, median (IQR), $	9567 (5728-17 096)	8104 (4767-13 196)	.02
Length of stay, median (IQR), d	5 (3-8)	5 (3-7)	.26
At 31-90 d			
Readmission	1451 (4.7)	122 (6.9)	.01
Readmission mortality	50 (3.4)	NA	NA
Cost, median (IQR), $	8917 (5358-16 598)	9379 (5013-14 111)	.79
Length of stay, median (IQR), d	4 (3-7)	4 (2-6)	.57
Ostomy closure	5983 (19.4)	315(17.8)	.30

Abbreviations: HP, Hartmann procedure; NA, not applicable; PAPD, primary anastomosis and diverting loop ileostomy.

^a^ Cell sizes with 10 or fewer patients were censored and marked NA in accordance with Nationwide Readmissions Database requirements.

^b^ Prolonged ventilation was defined as more than 96 hours.

**Figure 1.**
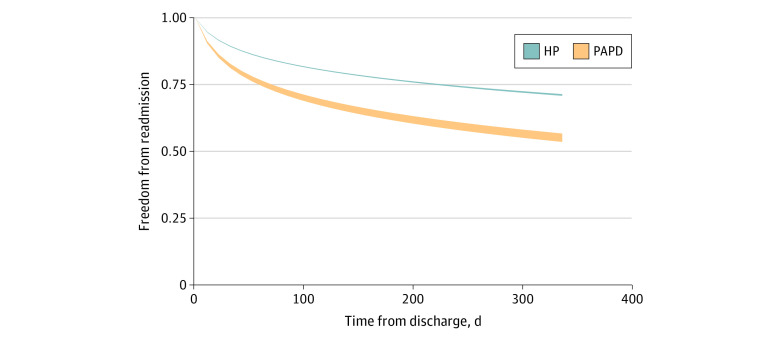
Probability of Freedom From Readmission by Operative Approach Risk-adjusted freedom from nonelective readmission adjusting for patient and hospital characteristics using inverse probability treatment weights and Royston-Parmar flexible parametric models is presented.

Among the estimated 32 333 patients in the entire IPTW operative cohort who survived to discharge, 18 459 IPTW survey-weighted patients (57.1%) who were readmitted, compared with 13 874 patients (42.9%) who were not readmitted, were older (median [IQR] age, 65 [55-76] years vs 62 [53-72] years; *P* < .001) but had decreased rates of heart failure (1133 patients [6.1%] vs 1016 patients [7.3%]; *P* = .01) (eTable 4 in the Supplement). While there was no statistically significant difference in index outcomes, PAPD was associated with an increased risk-adjusted hazard of unplanned readmissions (hazard ratio [HR], 1.79; 95% CI, 1.44-2.23) (Figure 1), with an increased rate of patients who underwent PAPD compared with those who underwent HP readmitted at least twice within the remainder of the calendar year (497 of 2003 patients [24.8%] vs 4471 of 30 330 patients [14.7%]; *P* < .001).

For patients undergoing PAPD vs patients undergoing HP, overall median (IQR) hospitalization costs were comparable for the remainder of the year ($87 484 [$57 979-$142 394] vs $87 084 [$55 508-$142 638]; *P* = .87) but median (IQR) total hospitalization days were not (13 [10-20] days vs 15 [12-22] days; *P* < .001). However, median (IQR) cost for the first readmission episode within 30 days of discharge was increased for patients undergoing HP ($13 428 [$8962-$19 623] vs $8500 [$5996-$12 589]; *P* < .001).Counterfactual modeling suggested that were all patients who underwent HP readmitted at the rate of patients who underwent PAPD within 1 month of discharge, readmission costs would increase by $3.7 million at the national level.

**Figure 2.**
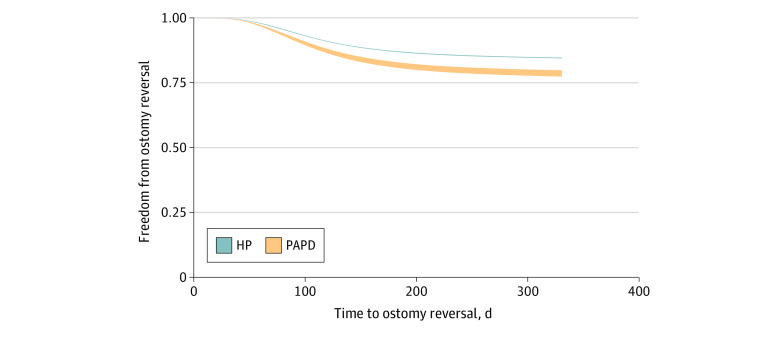
Probability of Freedom From Ostomy Reversal by Operative Approach Risk-adjusted hazard of ostomy closure adjusting for patient and hospital characteristics using inverse probability treatment weights and Royston-Parmar flexible parametric models is presented.

Among 3752 unplanned readmissions within 30 days, there were 2217 patients (59.1%) readmitted for bowel obstruction, peritonitis, or infectious or kidney complications. Among 336 patients who underwent PAPD who were readmitted within 30 days, compared with 3416 patients who underwent HP who were readmitted within 30 days, there were statistically significantly increased rates of kidney failure (71 patients [21.1%] vs 86 patients [2.5%]; *P* < .001) but not infectious complications (80 patients [23.8%] vs 898 patients [26.3%]; *P* = .54) or obstruction (48 patients [14.3%] vs 530 patients [15.5%]; *P* = .65). The unadjusted overall number and rate of stoma closures over subsequent readmissions was 6298 of 32 623 patients (19.3%) within the remainder of the year. However, PAPD was associated with increased hazard of ostomy reversal compared with HP (HR, 1.46; 95% CI, 1.08-1.99) (Figure 2). Furthermore, median (IQR) days to stoma closure was decreased for patients who underwent PAPD (65 [47-94] days) compared with the HP cohort (111 [86-160] days; *P* < .001).

## Discussion

Emergent surgical management of diverticular disease has evolved, with increasing application of resection and primary anastomosis to decrease subsequent operative morbidity of reversal. Given the current controversies surrounding the relative benefits of diverting loop ileostomy vs the traditional HP, this cross-sectional study investigated a nationwide cohort of patients with complicated diverticulitis and made several important observations. First, use of PAPD increased over the study period and was used among more than 7% of patients requiring diversion in 2017. Undergoing PAPD was associated with comparable mortality with HP after adjusting for differences in baseline characteristics using IPTW. Despite a more favorable risk profile, patients who underwent PAPD exhibited similar rates of adverse events and nonhome discharge. After index discharge, patients receiving PAPD incurred more frequent unplanned readmissions but had a higher hazard of ostomy reversal. Several of these findings deserve further discussion.

In the setting of acute perforation, fecal spillage, and possible hemodynamic instability, anastomotic breakdown is a feared complication associated with colonic resection with primary anastomosis. Protective proximal diversion may be associated with a decrease in incidence and severity of leaks and thus improved safety of primary anastomotic techniques. In contrast to HP, which warrants a more extensive operation to reverse treatment, PAPD has been proposed as an alternative with potentially increased reversal rates. Of 3 early randomized clinical trials examining outcomes associated with HP and PAPD, 2 were stopped prematurely owing to difficulty with enrollment or safety concerns during interim analysis.^7,8^ Published studies^8,9^ have found increased stoma reversal rates with PAPD but similar acute safety profiles compared with HP. Taken together, the authors of these randomized clinical trials advocated for increased use of PAPD. Using IPTW analysis accounting for differences in baseline characteristics, we found that PAPD and HP had comparable odds of acute complications and inpatient mortality. Our findings are in agreement with those of Lee et al,^24^ who used the NSQIP database and found comparable index outcomes associated with HP and PAPD. Overall, our findings suggest the relative safety profile of PAPD vs HP among carefully selected patients without a clear clinical advantage compared with HP during index hospitalization.

Consistent with the available literature, patients undergoing PAPD were significantly younger, with fewer major comorbidities, such as heart failure and obstructive pulmonary disease. Furthermore, patients undergoing HP more commonly had markers associated with significant end-organ dysfunction. It is not surprising that patients requiring operative intervention within the first 24 hours, with coagulopathy, chronic steroid use, and peritonitis, were unlikely to be selected for primary anastomosis. Rigorous risk-adjustment with IPTW suggests the association of selection bias with treatment strategy, which should be considered when comparing index mortality between HP and PAPD in our analysis and the available literature.

Differences in acute and chronic comorbidities suggest the importance of tailored patient selection. Furthermore, the association between age and acuity may result in similar patient severity in an older patient with decreased physiologic reserve and a younger patient with delayed presentation. Surgeons have achieved comparable outcomes with use of PAPD through rigorous patient selection. Taken together, our results suggest a cautious approach to routine adoption of PAPD in practice guidelines.

Beyond patient comorbidities, private insurance coverage was associated with increased use of PAPD. Insurance status, which has been commonly considered a marker associated with socioeconomic status and access to health care resources, further suggests the intrinsic differences in these populations.^25^ In our study, the rate of PAPD use was similar across teaching and nonteaching hospitals. Hospital annual diverticultitis admission volume was not associated with rate of PAPD. To our knowledge, these hospital characteristics have not previously been examined extensively given that most of the randomized clinical trials had some level of hospital homogeneity. While the most recent NSQIP analysis did not comment on hospital teaching status, an antecedent National Inpatient Sample analysis from 1998 to 2011 found an increased rate of proximal diverting ileostomy at teaching institutions.^24,26^ The observed similar rate of PAPD at teaching and nonteaching hospitals in our study may be associated with practice changes in response to society guidelines that have led to expansion of this surgical technique beyond academic settings. Interestingly, PAPD was less commonly used at hospitals considered high performers based on observed to expected sigmoidectomy operative mortality. Whether this observation is associated with increased acuity that is not captured in the database may warrant further investigation.

Importantly, the surgical equipoise between HP and PAPD suggested by available data may be associated with surgeon experience and comfort. An early observational study by Resio et al^3^ used the 2005 to 2015 NSQIP data set and found that PAPD was associated with increased risk of reoperation among patients with emergent conditions. A study^14^ using the New York state all-payer database over a 14-year period found that specialized training in colorectal surgical procedures was associated with decreased rates of mortality and complications after HP and PAPD. Given that most operations are performed by noncolorectal-trained surgeons, adoption of PAPD in the emergent setting should remain deliberate. Available evidence supports judicious use of PAPD despite recommendation from professional societies advocating for its more liberal application.

While 2 studies^3,27^ comparing HP with PAPD characterized rates of ostomy reversal, few studies have directly compared rates of unplanned readmissions, to our knowledge. Despite a decreased rate of comorbidities and younger age among patients undergoing PAPD, we found that these patients had significantly more frequent readmissions on raw and adjusted analyses. Consistent with expansive literature on ileostomy readmissions, patients who underwent PAPD were more likely to require rehospitalization for electrolyte abnormalities and acute kidney injury.^28,29,30^ Comparing these 2 modalities, the overall rate of ostomy reversal in our study was increased among patients who underwent PAPD compared with those who underwent HP.^8,9,10^ Numerous factors are associated with ostomy reversal, such as patient comorbidities, occurrence of postoperative complications, patient race, and insurance status.^13,31,32^ The discrepancy in the observed ostomy reversal rate in our study vs that in available literature may be associated with the above factors, as well as limitations of the NRD.

### Limitations

This study has several important limitations. The NRD lacks granular data, such as radiographic, physiologic, and laboratory parameters. Nutritional parameters and duration of symptoms, which are important clinical considerations when selecting operative strategy, are unavailable. We limited our analysis to patients who underwent surgical intervention within the first 48 hours of hospitalization to decrease heterogeneity and to select for the cohort with the most acute illness. Furthermore, changes in administrative coding schema from *ICD-9* to *ICD-10* in 2015 limit the interpretation of our trend analysis, particularly the magnitude of increase in PAPD performance. Nuances of intraoperative decision-making on feasibility and success of primary anastomosis, such as extent of colonic wall thickening and hemodynamic instability, are not accounted for in this study. Given that patient identifiers are not retained across years within the NRD, we were unable to follow up patients beyond the same calendar year, thus underestimating readmissions and reversal rates. Nonetheless, the interval to ostomy closure does not vary based on diversion strategy and should not significantly alter our findings. Additionally, we are unable to adequately capture out of hospital mortality, a clearly important end point. Despite these limitations, we used a nationally representative sample of patients and robust statistical methods to decrease potential bias and enhance the generalizability of our findings.

## Conclusions

This cross-sectional study found that PAPD was infrequently used and associated with comparable risk-adjusted mortality and complications vs HP. Patients with PAPD were more likely to be rehospitalized within 90 days after discharge and had increased risk-adjusted hazard for ostomy reversal. Our study provides a nationally representative, inclusive, and pragmatic perspective on diversion strategies in the treatment of complicated diverticulitis. The relatively favorable risk profile of patients undergoing PAPD in our study suggests the need for meticulous selection to achieve favorable outcomes. These findings suggest that data regarding increased rates of readmission and risk of ostomy reversal should be discussed with patients in order to better inform shared decision-making.

## Notes

Supplement. **eTable 1.** Administrative Codes Used for Definition of Multiorgan Dysfunction and Complications **eTable 2.** Administrative Codes Used for Cohort Identification **eTable 3.** Patient and Hospital Demographics by Operative Approach After Application of Inverse-Probability Treatment Weights **eTable 4.** Patient and Hospital Characteristics Associated With Readmission **eFigure.** Study Consort Diagram
