# Supplementary material for: Comparison of Diversion Strategies for Management of Acute Complicated Diverticulitis in a US Nationwide Cohort
Source: JAMA Netw Open. 2021 Nov 5;4(11):e2130674. doi: 10.1001/jamanetworkopen.2021.30674 (PMC8571654; doi:10.1001/jamanetworkopen.2021.30674)
Supplement: Supplement. — eTable 1. Administrative Codes Used for Definition of Multiorgan Dysfunction and Complications eTable 2. Administrative Codes Used for Cohort Identification eTable 3. Patient and Hospital Demographics by Operative Approach After Application of Inverse-Probability Treatment Weights eTable 4. Patient and Hospital Characteristics Associated With Readmission eFigure. Study Consort Diagram [file jamanetwopen-e2130674-s001.pdf]

Supplemental Online Content

Sanaiha Y, Hadaya J, Aguayo E, Chen F, Benharash P. Comparison of diversion strategies for management of acute complicated diverticulitis in a US nationwide cohort. *JAMA Netw Open*. 2021;4(11):e2130674. doi:10.1001/jamanetworkopen.2021.30674

eTable 1. Administrative Codes Used for Definition of Multiorgan Dysfunction and Complications

eTable 2. Administrative Codes Used for Cohort Identification

eTable 3. Patient and Hospital Demographics by Operative Approach After Application of Inverse-Probability Treatment Weights

eTable 4. Patient and Hospital Characteristics Associated With Readmission

eFigure. Study Consort Diagram

This supplemental material has been provided by the authors to give readers additional information about their work.

© 2021 Sanaiha Y et al. *JAMA Network Open.*

eTable 1. Administrative Codes Used for Definition of Multiorgan Dysfunction and Complications

Diagnoses	ICD 9	ICD 10
Multi-organ dysfunction		
Hypotension	458x	I95x
Acute Respiratory Failure	518.82, 518.83, 518.8	J96x
Altered Mental Status	780.0, 780.09	R40x, 080x, 081x
Acute Kidney Injury	584.x, 586.x, 588.x, 585.x	N17x, N18x, N19x, N25x
Acute hepatic dysfunction	570.x, 571.9	K720x, K729x, K762x, K763x, K769x, K741x,
Thrombocytopenia	287.4, 287.49, 287.5	D6951x, D6959x, D696x
Composite Complications		
Stroke	997.02, 433.01, 433.11, 433.21, 433.31, 433.81, 433.91, 434.01, 434.11, 434.91, 437.0, 437.1, 437.4, 437.5, 437.7, 437.9	I6322, I63139, I63239, I63019, I63119, I63219, I6359, I6320, I6330, I6340, I6350, I672, I6781, I6782, I6789, I677, G454, I679
MI	410.00, 410.01, 410.10, 410.11, 410.20, 410.21, 410.30, 410.31, 410.41, 410.50, 410.51, 410.60, 410.61, 410.81, 410.91	I2101, I2109, I2119, I2200, I2210, I2111
Cardiac Arrest	427.5	I46
Pneumonia	481, 482.1, 482.2, 482.31, 482.32,482.39, 482.41, 482.42, 482.49, 482.81, 482.82, 482.83, 482.84, 482.89, 482.9, 997.3, 997.32, 997.39	J13, J14, J181, J150, J151, J15211, J15212, J154, J153, J152, J158, J155, J156, A481, J159, J95851, J9589, J95859, J9588
Acute Respiratory Failure	518.5	J96
Pulmonary Edema	518.4	J810
Deep Vein Thrombosis	451.1, 451.2, 451.81, 451.9, 453.2, 453.40, 453.41, 453.42, 453.8, 453.9	I8010, I80209, I80219, I803, I809, I82220, I82409, I82419, I82429, I82439, I824Y9, I82449, I824Z9, I82619, I82629, I82609, I82A19, I82B19, I82C19, I82290, I82890, I8291
Prolonged Mechanical Ventilation	967.2	5A1955Z, 5A1945Z
Urinary tract infection	599.0	N390
Wound Infection	998.51, 998.59	T814XXA, K6811
Sepsis	038, 038.1, 038.11, 038.12, 038.19, 0382, 0383, 0384, 038.41, 038.42, 038.43, 038.44, 038.49, 038.8	A409, A412, A4101, A4102, A411, A403, A414, A4150, A413, A4151, A4152, A4153, A4159, A4189, A419, R6520, T80219A, T80211A, T80212A, T8022XA, T8029XA
Septicemia	038.9	A419

© 2021 Sanaiha Y et al. *JAMA Network Open.*

eTable 2. Administrative Codes Used for Cohort Identification

Inclusion Criteria	ICD 9	ICD 10
Diverticulitis	562.11, 562.13	K5720, K5721, K5732, K5733, K5740, K5741, K5752, K5753, K5792, K5793, K5780, K5781
Sigmoidectomy	45.75, 45.76	0DTN0ZZ, 0DTN4ZZ, 0DTM0ZZ, 0DTM4ZZ, 0DBN0ZZ, 0DBN4ZZ
Ileostomy	46.20, 46.21, 46.22, 46.23	0D1B0Z4, 0D1B4Z4, 0D1B8Z4
Colostomy	46.10, 46.11, 46.13	0D1L0Z4, 0D1L4Z4, 0D1N0Z4, 0D1N4Z4, 0D1M0Z4, 0D1M4Z4, 0D1N0Z4, 0D1N4Z4
Operative Stratification	Codes for:	
Hartmann's Procedure	Sigmoidectomy and colostomy without ileostomy	
Primary Anastomosis with Proximal Diversion	Sigmoidectomy and ileostomy without colostomy	
Sigmoidectomy	Sigmoidectomy without ileostomy or colostomy	
Excluded Sigmoidectomy	Sigmoidectomy with both ileostomy and colostomy	

© 2021 Sanaiha Y et al. *JAMA Network Open.*

eTable 3. Patient and Hospital Demographics by Operative Approach After Application of Inverse-Probability Treatment Weights

	HP	PAPD	*P Value*
	**N=31,562***	**N=2,057***	
Age (median years, IQR)	62 (52-72)	63 (55-74)	0.062
Female (%)	16,242 (51.5)	1,066 (51.8)	0.91
Congestive Heart Failure (%)	2,309 (7.3)	176 (8.6)	0.46
Coronary Artery Disease (%)	3,357 (10.6)	217 (10.6)	0.97
Chronic Pulmonary Parenchymal Disease (%)	4,915 (15.6)	273 (13.3)	0.24
Pulmonary Hypertension (%)	2,130 (6.8)	108 (5.3)	0.19
Peripheral Vascular Disease (%)	1,561 (4.9)	84 (4.1)	0.37
Hypertension (%)	15,479 (49.0)	1,102 (53.6)	0.16
Diabetes (%)	4,074 (12.9)	218 (10.6)	0.19
Chronic Renal Dysfunction (%)	2,203 (7.0)	129 (6.3)	0.68
Chronic Liver Disease (%)	1,123 (3.6)	65 (3.1)	0.58
Coagulopathy (%)	1,353 (4.3)	57 (2.8)	0.07
Multi-organ dysfunction (%)	10,450 (33.1)	654 (31.8)	<0.001
Obesity (%)	5,177 (16.4)	321 (15.6)	0.70
Weight Loss (%)	4,214 (13.4)	267 (13.0)	0.87
Electrolyte Abnormalities (%)	12,968 (41.1)	840 (40.8)	0.94
Peritonitis (%)	5,977 (18.9)	490 (23.8)	0.09
Ascites (%)	1,263 (4.0)	65 (3.2)	0.23
Chronic Steroid Use (%)	948 (3.0)	63 (3.0)	0.98
Percutaneous drain (%)	1,297 (4.1)	88 (4.3)	0.83
Laparoscopy (%)	1,101 (3.5)	31 (1.5)	0.002
Laparoscopic converted to open (%)	797 (2.5)	104 (5.0)	0.02
Income Quartile			
0-25th (%)	6,947 (22.3)	406 (20.1)	0.46
25-50th (%)	8.338 (26.7)	631 (31.2)	
50-75th (%)	8,105 (26.0)	495 (24.5)	
75-100th (%)	7,785 (25.0)	488 (24.2)	
Private Insurer (%)	12,787 (40.5)	740 (36.0)	0.25
Teaching Status			0.36
Metropolitan, non-teaching (%)	8,817 (28.0)	791 (38.5)	<0.001
Metropolitan, teaching (%)	20,445 (64.8)	931 (45.3)	
Rural (%)	2,300 (7.3)	335 (16.3)	
Safety-Net Hospital Status (%)	6,904 (21.9)	552 (26.8)	0.04
Inter-hospital Transfer (%)	470 (1.5)	27 (1.4)	0.83
Annual Sigmoidectomy Volume (Median, IQR)	37 (20-63))	17 (8-37)	<0.001
Annual Diverticulitis Volume (Median, IQR)	178 (108-267)	116 (53-181)	<0.001

*Survey-weighted estimates using inverse probability treatment weights are discrepant compared to non-IPTW estimates given modifications of discharge weights according to inverse probability treatment weights

© 2021 Sanaiha Y et al. *JAMA Network Open.*

eTable 4. Patient and Hospital Characteristics Associated With Readmission

	Non-Readmitted	Readmitted	*P Value*
	**N=13,874**	**N=18,459**	
Age (median years, IQR)	62 (53-72)	65 (55-76)	
Female (%)	7,344 (52.9)	9,155 (49.6)	0.002
Congestive Heart Failure (%)	1,016 (7.3)	1,133 (6.1)	0.01
Coronary Artery Disease (%)	1,388 (10.0)	2,001 (10.8)	0.14
Chronic Pulmonary Parenchymal Disease (%)	2,107 (15.2)	2,834 (15.4)	0.81
Pulmonary Hypertension (%)	925 (6.7)	1,259 (6.8)	0.79
Peripheral Vascular Disease (%)	749 (5.4)	772 (4.2)	0.007
Hypertension (%)	6,870 (49.5)	9,156 (49.6)	0.94
Diabetes (%)	1,776 (12.8)	2,309 (12.5)	0.65
Chronic Renal Dysfunction (%)	944 (6.8)	1,184 (6.4)	0.43
Chronic Liver Disease (%)	496 (3.6)	550 (3.0)	0.10
Coagulopathy (%)	575 (4.1)	611 (3.3)	0.01
Multi-organ dysfunction (%)	4,575 (33.0)	5,399 (29.2)	<0.001
Obesity (%)	2,381 (17.2)	3,042 (16.5)	0.42
Weight Loss (%)	2,061 (14.9)	2,048 (11.1)	<0.001
Electrolyte Abnormalities (%)	5,685 (41.0)	7,257 (39.3)	0.11
Peritonitis (%)	2,423 (17.5)	3,731 (20.2)	0.002
Ascites (%)	554 (4.0)	636 (3.4)	0.15
Chronic Steroid Use (%)	409 (2.9)	567 (3.1)	0.82
Percutaneous drain (%)	559 (4.0)	759 (4.1)	0.86
Laparoscopy (%)	455 (3.3)	663 (3.6)	0.54
Laparoscopic converted to open (%)	395 (2.8)	498 (2.7)	0.69
Income Quartile			
0-25th (%)	3,300 (24.1)	3773 (20.7)	0.001
25-50th (%)	3,680 (27.0)	4,926 (27.0)	
50-75th (%)	3,421 (25.0)	4,834 (26.5)	
75-100th (%)	3,270 (24.0)	4,720 (25.9)	
Private Insurer (%)	4,882 (35.2)	8,421 (45.6)	<0.001
Teaching Status			
Metropolitan, non-teaching (%)	3,873 (27.9)	5,363 (29.0)	0.04
Metropolitan, teaching (%)	8,878 (63.3)	11,743(63.6)	
Rural (%)	1,212 (8.7)	1,352 (7.3)	
Safety-Net Hospital Status (%)	3,343 (24.1)	3,842 (20.8)	<0.0001
Inter-hospital Transfer (%)	230 (1.7)	214 (1.2)	0.04
Annual Sigmoidectomy Volume (Median, IQR)	35 (19-61)	36 (19-61)	0.20
Annual Diverticulitis Volume (Median, IQR)	170 (103-261)	178 (105-267)	0.02
Primary anastomosis proximal diversion (%)	624 (4.5)	1,379 (7.5)	<0.003

© 2021 Sanaiha Y et al. *JAMA Network Open.*

eFigure. Study Consort Diagram

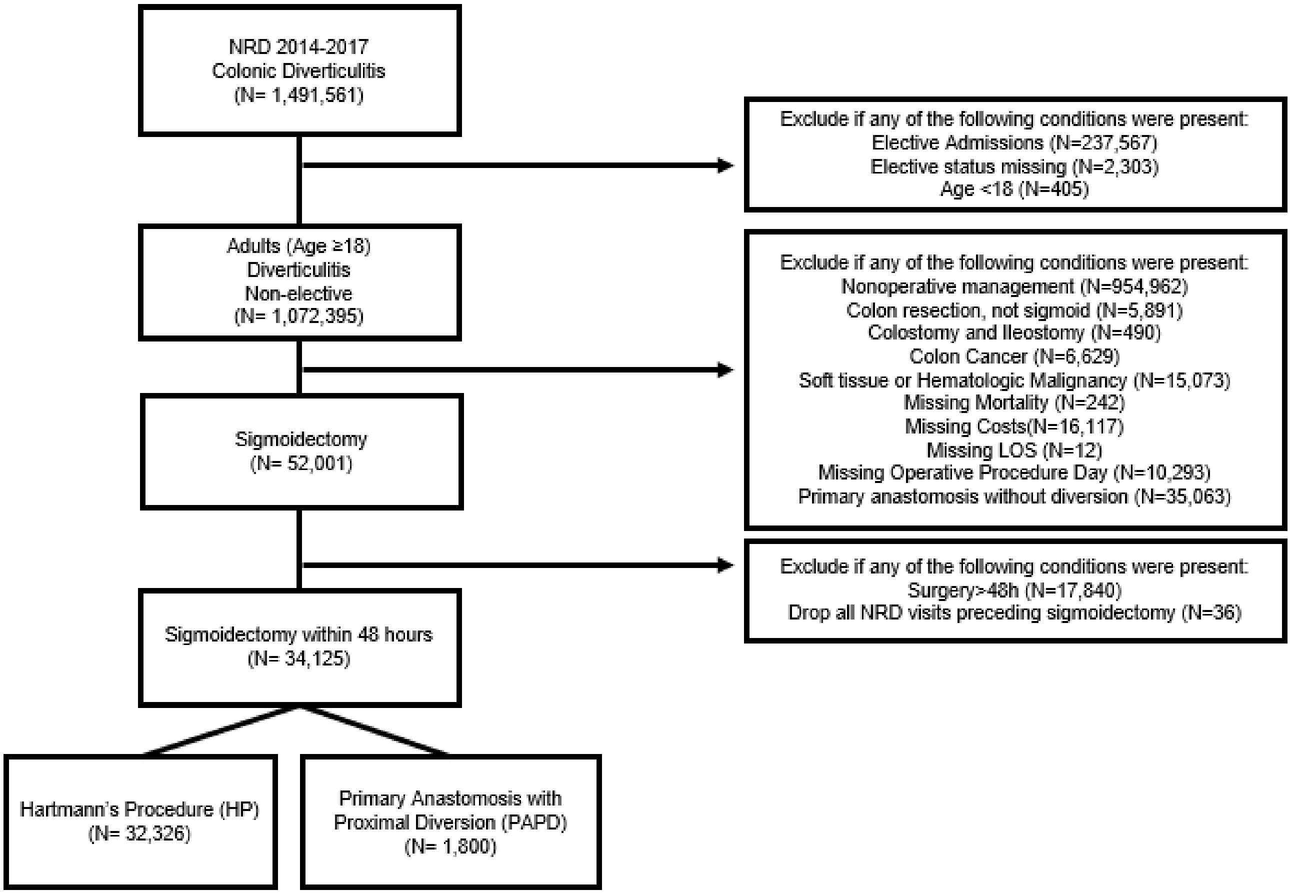

© 2021 Sanaiha Y et al. *JAMA Network Open.*
